# “First AID” for T cell adoptive transfer: TCR affinity maturation by somatic hypermutation

**DOI:** 10.1186/2051-1426-1-S1-P11

**Published:** 2013-11-07

**Authors:** Yosi M Gozlan, Esther Tzehoval, Lea Eisenbach

**Affiliations:** 1 Immunology, Weizmann Institute of Science, Rehovot, Israel

## 

Adoptive cell transfer (ACT) of tumor specific T lymphocytes, after host immunodepletion, was shown to mediate objective cancer regression of metastatic melanoma. Effective T cell activation depends, among other factors, on the functional avidity of the peptide-MHC complex (pMHC) to the T cell receptor (TCR), i.e. on the affinity and the number of pMHC-TCR contacts. Since the T cell repertoire is controlled by negative and positive selection in the thymus, naturally occurring TCRs have mostly low affinities, in the range of 1-100µM. Moreover, unlike antibodies whose affinities improve over time by somatic hypermutation (SHM), TCR do not undergo SHM. In our research we developed a system that can increase the affinity of a TCR to its ligand by subjecting TCR genes to somatic hypermutation, directed by the mutator enzyme Activation Induced cytidine Deaminase (AID). Affinity maturation reactions are performed ex-vivo in easy transfectable cells and affinity maturated TCRs are selected by tetramer staining followed by FACS sorting. The affinity maturation system is designed to be modular so the maturation can be done in several cycles in order to optimize TCR affinity. The affinity maturated TCRs are used to create anti-tumor reactive T cells by means of gene transfer into naïve lymphocytes or anti-viral CTL. These transformed T cells are then functionally tested in-vitro and in-vivo with murine melanoma models. Using this system we are trying to define the parameters that govern the changes in affinity as well as the biological consequences of these changes. Such systems can potentially be used to augment T cell responses to low immunogenic Tumor Associated Antigen (TAA) peptides, for the treatment of cancer.

